# Discovery of candidate genes for nonsyndromic cleft lip palate through genome-wide linkage analysis of large extended families in the Malay population

**DOI:** 10.1186/s12863-016-0345-x

**Published:** 2016-02-11

**Authors:** Nurul Syazana Mohamad Shah, Iman Salahshourifar, Sarina Sulong, Wan Azman Wan Sulaiman, Ahmad Sukari Halim

**Affiliations:** Reconstructive Science Unit, School of Medical Sciences, Universiti Sains Malaysia, Kelantan, Malaysia; Department of Biology, School of Basic Sciences, Science and Research Branch, Islamic Azad University, Isfahan, Iran; Human Genome Center, School of Medical Sciences, Universiti Sains Malaysia, Kelantan, Malaysia; School of Medical Sciences, Health Campus, Universiti Sains Malaysia, Kelantan, Malaysia

**Keywords:** Nonsyndromic cleft lip palate, Large families, Microarray, Genome-wide linkage analysis, Candidate genes

## Abstract

**Background:**

Nonsyndromic orofacial clefts are one of the most common birth defects worldwide. It occurs as a result of genetic or environmental factors. This study investigates the genetic contribution to nonsyndromic cleft lip and/or palate through the analysis of family pedigrees. Candidate genes associated with the condition were identified from large extended families from the Malay population.

**Results:**

A significant nonparametric linkage (NPL) score was detected in family 100. Other suggestive NPL and logarithm of the odds (LOD) scores were attained from families 50, 58, 99 and 100 under autosomal recessive mode. Heterogeneity LOD (HLOD) score ≥ 1 was determined for all families, confirming genetic heterogeneity of the population and indicating that a proportion of families might be linked to each other. Several candidate genes in linkage intervals were determined; *LPHN2* at 1p31, *SATB2* at 2q33.1-q35, *PVRL3* at 3q13.3, *COL21A1* at 6p12.1, *FOXP2* at 7q22.3-q33, *FOXG1* and *HECTD1* at 14q12 and *TOX3* at 16q12.1.

**Conclusions:**

We have identified several novel and known candidate genes for nonsyndromic cleft lip and/or palate through genome-wide linkage analysis. Further analysis of the involvement of these genes in the condition will shed light on the disease mechanism. Comprehensive genetic testing of the candidate genes is warranted.

## Background

Orofacial clefts are congenital malformation that comprises of a large fraction of human birth defects that affects the lip and/or palate. Orofacial clefts are one of the most common human congenital disorder reported in Western countries and second most common birth defects among newborn [1, 2]. It arises approximately 1 per 500 to 1000 live births with ethnic and geographic variation [1, 3]. Asian and Native North American population has highest birth prevalence by 2 per 1000 births compared to the other populations [4, 5]. In Malaysia, the rate of occurrence of cleft was 1.24 per 1000 livebirths or 1.20 per 1000 deliveries while the latest reported the incidence of 1 in every 700 newborn babies had a cleft lip and/or palate condition [6, 7]. It was also reported that the highest incidence of clefts was among Chinese with 1.9 per 1000 deliveries while the Malays had the lowest incidence with 0.98 per 1000 deliveries [6].

Clefts can be categorized into syndromic and isolated forms, according to whether affected individuals have other physical and developmental anomalies. The focus of this study was on nonsyndromic clefts. Nonsyndromic cleft lip with or without palate (NSCLP) is a malformation disease that does not show any other signs or symptoms of abnormal condition such as abnormal physical appearance or psychological disorder. Nonsyndromic cases for both cleft lip with or without cleft palate are heterogenous with multifactorial etiology, in which both genetic and environmental factors take place [2]. Contribution of both genetic and environmental factors to the development of orofacial clefts making the causative mechanism is complicated to elucidate [8]. A discovery of genetic transmission of cleft in the family was highlighted in an effort to identify clefting genes in this study. To date, there has been a lot of studies discovering multiple causative genes that cause the nonsyndromic cleft lip palate (NSCLP) formation [9, 10].

Linkage analysis of multiplex families and association studies using either case–control or family-based designs has become primary methods in identifying potential genes for CLP [11]. The first linkage study was done by Beiraghi et al., [12] that reported the possible gene in 4q region tested on a single five-generation family of cleft lip palate but later reported to have no significant evidence for linkage in 56 families tested [13]. Linkage studies have revealed several candidate regions studied in different populations. A consanguineous family with 17 members has reported to have homozygous 237-kb deletion at locus 1p31 among the family members that cause cleft lip [14]. Three regions of chromosome 1; 1p36, 1q21 and 1q32-42.3, which 1p36 regions had positive scores by parametric linkage approach and pairwise analysis found susceptibility gene on 1q21 and 1q32-42.3 regions in 38 families of NSCLP from Northeastern Italy [15]. Regions on chromosomes 6p, 2p, 4q and 17q have all shown some evidence of linkage to NSCLP [16].

Even after many years of carrying out investigation, the mode of inheritance of CLP remains controversial and challenging that were probably due to the samples and models employed [16]. Linkage analyses are tools for mapping a disease gene on a chromosome, and they involve the use of genetic markers [16]. Studies of susceptible linkage genes using microarray platform has led to a significant number of new biological discoveries and important correlations between gene expression pattern and disease states [17]. Here, we presented the results of genome-wide linkage analysis on 8 multiplex families with positive family history of NSCLP.

## Methods

### Subjects

Eight large extended families with 91 individuals were included, comprised of ≥3 affected members in each family. The blood withdrawal was undertaken either by appointment at clinic or home visit. All individuals have been referred to the craniofacial specialist. This study was approved by the Research Ethics Committee (Human) of the Universiti Sains Malaysia, Health Campus, Malaysia; Reference No.: USMKK/PPP/JEPeM [258.3.(3)] and written informed consent for blood withdrawal was obtained from the participants or their parent for the patients below 16 years old. Informed consent for publication permission submitted nationally or internationally with regards to any data output, personal information or family background was also obtained from all the participants or their parent for the patients below 16 years old.

### Inclusion and exclusion criteria

All families either having cleft lip (CL), cleft lip and palate (CLP) or cleft palate (CP) were all included in this study. Patients were screened by specialists of plastic surgery for any major abnormalities or syndromes. Patients with minor anomaly such as low-set ears, hypertelorism, clinodactyly and single palmar crease were included. Meanwhile, if the patients have a heart disease, any syndromes associated with cleft, or a major or more than two minor defects, they would be excluded from this study.

### DNA extraction for blood samples

Genomic DNA was extracted from 200 μl of heparinized peripheral blood using QIAamp DNA Mini Kit (Qiagen, USA) following the manufactures instruction. The quality and quantity of DNA samples were quantified using Infinite® 200 PRO NanoQuant (Tecan). The concentration was measured at absorbance of 260/280 nm. Purity was determined by calculating the ratio of absorbance at 260 nm to absorbance at 280 nm. Pure DNA has an A_260_/A_280_ ratio of 1.7–1.9.

### Microarray analysis

Extracted DNA from eight large extended families was used for microarray analysis using Illumina Infinium Human Linkage-24 Beadchip. All the samples were genotyped at St George’s Hospital, University of London.

On day 1, Standard DNA plate (250 ng/μl) and blank (0 ng/μl) DNA were prepared and the quantitated DNA was read with Infinium LIMS Database. After that, DNA samples were denatured and neutralized with 4 μl 0.1 N NaOH followed by vortex the plate at 1600 rpm for 1 min and centrifuge to 280 xg for 1 min. The plate was incubated overnight in the Illumina Hybridization Oven at 37 °C.

On Day 2, DNA were fragmented and precipitated. Precipitated DNA then were incubated in Illumina Hybridization Oven for 1 h at 48 °C. After that, the resuspended DNA samples were dispensed onto BeadChips and incubated into the 48 °C Illumina Hybridization Oven for at least 16 h.

On Day 3, BeadChips were prepared for staining process. After washing and submerged in PB1, labeled nucleotides were added to DNA to extend the primers. The primers then were stained using a sandwich-staining protocol followed by disassembled the Flow-Through Chambers and coated the BeadChips for protection. Image of the BeadChip were scanned using iScan Reader. Data from the scanned images were analyzed using Illumina’s Genome Studio Genotyping Module. Markers with a minor allele frequency greater than 5 % and call rate higher than 95 % were used in the analyses. The data were converted to “.txt” file using Beadstudio program for further analysis by downstream software programs.

### Statistical analysis methods

#### Linkage analysis

Easy Linkage Plus v5.08 software was used to enable linkage analyses for large scale SNP data. It allows multipoint simulation studies as well as checking for Mendelian/non-Mendelian genotyping errors and Hardy-Weinberg equilibrium (HWE) [18]. Gene Hunter-Multipoint Linkage Analysis v2.1r5 was used to do multipoint parametric and non-parametric analysis. Chromosomal interval and spacing markers with 0.2 cM was used in this analysis.

Parametric analysis was carried out under both dominant and recessive mode of inheritance. A disease allele frequency of 0.001 and penetrance vectors of 0.99 for homozygous and heterozygous mutant was used for autosomal dominant. Similar allele frequency and penetrance parameters were used for homozygous mutant for autosomal recessive. Nonparametric analysis was carried out to evaluate allele sharing among affected individuals [19]. The outputs consist of chromosomal/genome-wide plots of LOD and NPL scores, *p* values and cM values. Plots display details of the used inheritance model, marker map, sex specific or sex-averaged marker positions. Plots were generated as ‘TOTAL’ plots averaging all families or plus individual family plots [18].

The thresholds for genome-wide linkage analysis were defined based on criteria proposed by Lander and Kruglyak [20]. Parametric LOD score indicates if LOD ≥ 1.9 but < 3.3 for suggestive linkage and ≥ 3.3 is indicating significant linkage. Meanwhile, the allele sharing NPL scores were categorized into three stages; score in between 2.2 and 3.5 is indicated as suggestive linkage, 3.6–5.3 indicates significant linkage and ≥ 5.4 indicates highly significant linkage respectively.

### Data quality control

Quality controls have been carried out to detect and eliminate errors in both genotype and pedigree data files. Phenotype error is detected when an affected individual misclassified as an unaffected individual or vice versa, which occurred due to sample mixing, inaccurate family information, misdiagnosis and wrong ID number in pedigree file and genotype file. Quality control is mandatory since phenotype errors can highly affect a value of LOD score.

### Check for Mendelian errors with pedcheck program

PedCheck is used to identify genotype incompatibilities in linkage analysis. It is essential to eliminate all the Mendelian inconsistencies in the pedigree data due to genotyping error or others. Mendelian errors was checked using PedCheck program version 1.1 [21]. PedCheck offers four error-checking algorithms in identification of an error.

On the first level, the nuclear-family algorithm uses the known genotypes to check for inconsistencies between parents and offspring. There is no genotype elimination in this level. At the second level, genotype elimination was performed via extended version of the Lange-Goradia algorithm. It uses the nuclear-family relationships to eliminate invalid genotypes in the pedigree. The third level Critical-Genotype algorithm and fourth level of Odds-Ratio algorithm attempts to identify any critical genotypes in the pedigree. The program will not move forward if detect level-1 errors. Therefore, level-1 errors need to be corrected.

### Detection of errors with merlin

In addition to PedCheck program, Merlin was used to detect for genotyping errors. Unlikely genotypes are equivalent to double recombinations in a short chromosomal segment. Unlikely genotypes were corrected by Pedwipe program together with Merlin package.

### Genetic map

Both Illumina 6K Linkage 24 deCODE Human Genetic Map and AFFY 100k Marshfield Human Sex-Averaged from Gene Hunter-Multipoint Linkage Analysis v2.1r5 were used for linkage analysis.

### Detection of chromosomal linkage interval and candidate genes

An output of linkage analysis was used to determine linkage interval of selected SNPs using National Center for Biotechnology Information (NCBI) web-based. The data from NCBI were transferred into Gene Distiller database. It is used to select and project genes from within a linkage interval, display gene specific information and sort the genes according to phenotype parameter [22].

## Results

### Family description

**Fig. 1:**
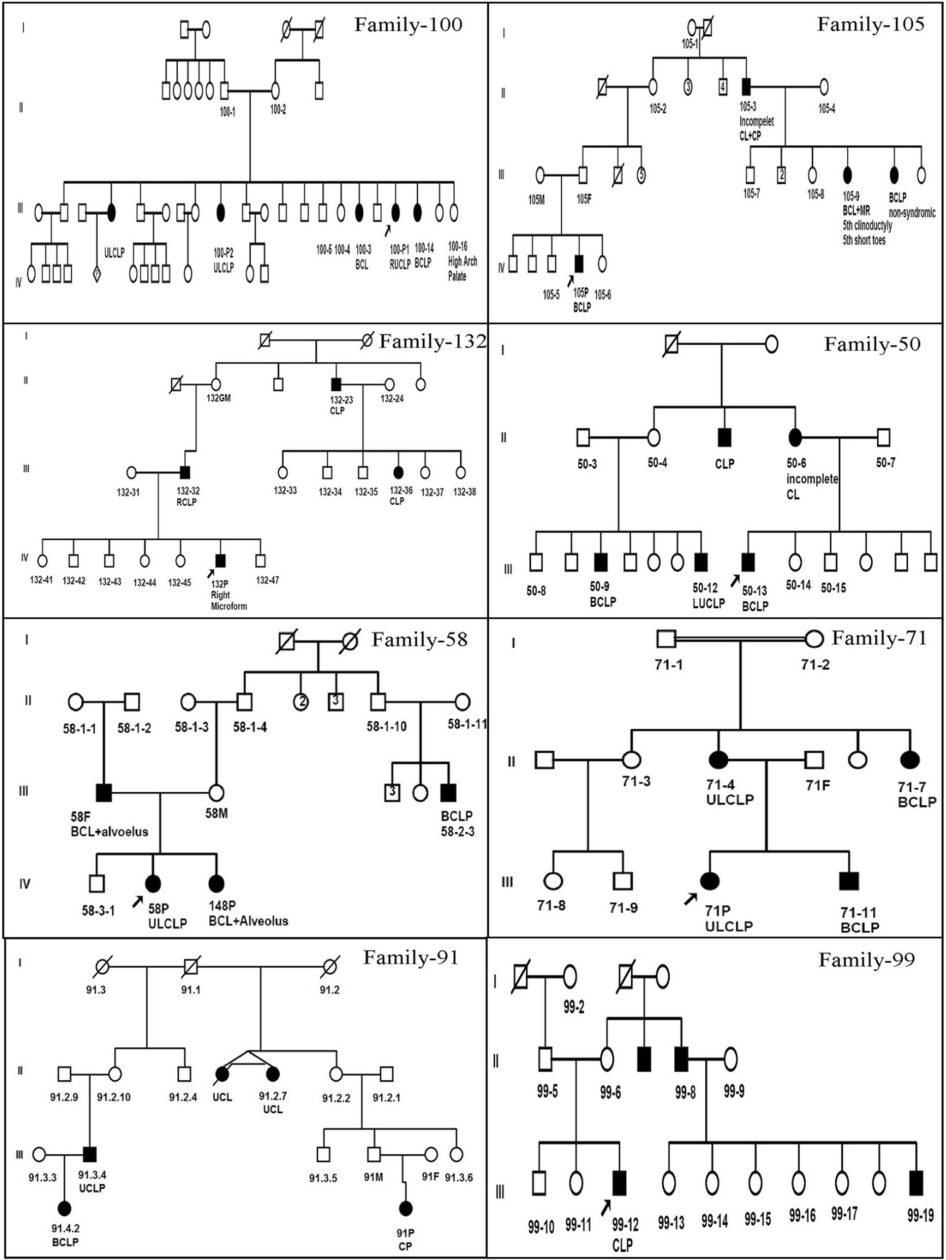
Family pedigree of three or four generations. Family tree of eight large extended families with orofacial clefts, including both affected and non-affected used in genome-wide linkage analysis

Eight large extended families consist of total 91 individuals were included. Size of each family was ranged between 9 and 14 individuals (Fig. 1). From the total, 30 individuals were found affected by oral clefts and another 61 individuals were not affected. Three families had five affected members while another five families had four affected members. One individual from family 105 had mental retardation with several minor dysmorphic features plus the presence of oral clefts, thus this individual was excluded from the study.

### Quality control evaluation

Prior to linkage analysis, unlikely genotypes and Mendelian inconsistencies were checked and eliminated using Merlin and Pedcheck programs. Pedcheck identified Mendelian inconsistencies in each family pedigree and level-1 errors. Pedigree files were corrected and Pedcheck was rerun until no more errors were detected. Merlin was used to check 5824 SNPs for a minimum call rate of 95 %. Uninformative and low call rate (<95 %) SNPs were eliminated from the analysis.

### Linkage analysis results

#### Nonparametric analysis

**Fig. 2:**
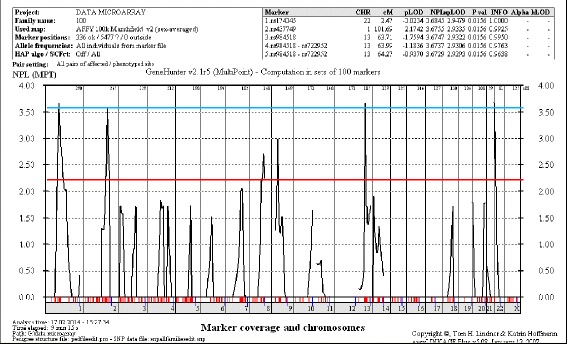
Genome-wide multipoint NPL plot for family 100. The suggestive threshold (NPL score between 2.20 and 3.50) at the genome-wide level is indicated by a red horizontal line. The significant threshold (NPL score > 3.60) is indicated by a blue horizontal line. Significant NPL scores between 3.67 and 3.68, with *p* < 0.05 on chromosome 1, 13 and 22 were obtained for family 100. This linkage data was analysed using Easy Linkage Plus v5.08 software implemented by Hoffmann and Lindner (2005)

All the subjects were genotyped using Illumina 6K Linkage 24 deCODE Human Genetic Map and the AFFY 100k Marshfield Human Sex-Averaged. All markers were in Hardy-Weinberg equilibrium (HWE) *p* value < 0.05. The overall call rate was > 98 % for all samples. Suggestive and significant linkage intervals were yielded with NPL score > 2 among the families using both genetic map. The output of total families has reached to the level of suggestive linkage for chromosomes 2q33.1-q35, 4q22.1-q25, 7q22.3-q33, and 14q12. However, by analyzing each family, family 100 was the only family that reached a significant linkage level with NPL scores of 3.67–3.68, *p* value < 0.05 (*p* = 0.02) on chromosome 1, 13 and 22. Meanwhile, other linkage intervals in family 50 and family 58 showed suggestive linkage as shown in Table 1 and Fig. 2.

**Table 1:** Linkage intervals with NPL score > 2 in each family determined using both the human genetic maps

Family	Chromosome	NPL	SNP	Position cM	Physical position
50	1p31.1-34.2	2.40	rs697590	82.65	42609867–61803889
	2q34-q36.3	2.49	rs1851328	213.99	211045482–227886773
	9q21.13-q22.2	2.47	rs2839899	75.60	77502160–93206737
58	4p15.2-p16.1	2.98	rs123043	31.84	8063501–25312357
	4q31.3-q33	2.04	rs4318612	158.9	154887604–170939829
	5p14.1-p15.33	3.15	rs13161353	41.15	601647–28733753
	5q14.1-q14.3	3.11	rs1566629	97.97	78829249–83307587
	6p11.2-12.3	3.17	rs1925154	80.06	56104522–56239724
	14q12	3.12	rs7160965	28.00	26751099–31733642
	16q12.1	2.56	rs1420533	63.60	52529714–52563626
**100**	**1p31.1-31.3**	**3.64**	rs400382	101.29	81898744–82364429
	**2q31.1-q35**	**3.68**	rs7602656	201.47	174680163–216195171
	**2q31.1-q35**	**3.69**	rs1002207	186.26	180689841–181554568
	**7q22.3-32.3**	**3.68**	rs1464890	130.88	106088278–131180918
	**8q22.1-q23.2**	**3.68**	rs2245832	101.69	94159583–111354022
	**11p15.3-p13**	**3.68**	rs4584574	38.59	11655268–31535355
	**13q22.2-q31.3**	**3.67**	rs722952	63.99	85656778–86230913
	**14q12-q21.1**	**3.68**	rs179562	26.78	24977594–38778344
	**22q11.1-q11.21**	**3.68**	rs174345	2.47	17553499–18033199

Significant NPL scores > 3.60 at various loci found in family 100 are highlighted in bold

### Parametric analysis

#### Recessive model

Under the recessive model, suggestive linkage peaks were obtained in family 99 at one linkage interval and in family 100 at six linkage intervals (Table 2). The genome-wide HLOD score was 2.63 (α = 0.122) in all families. Other chromosomal regions that attained HLOD ≥ 1 and α ≤ 0.71 were 1p31.1, 2q33.1-q35, 3q13, 4q23 and 11p14.1. These findings confirmed genetic heterogeneity in this population, indicating that a proportion of families may have a linkage. Of all families, family 100 was the only family in which several chromosomal regions attained a LOD ≥ 1.9 under the recessive model. No consanguineous marriage was detected in this family.

**Table 2:** Linkage intervals with LOD score > 2 in each family using both the Illumina 6K linkage 24 deCODE human genetic map and AFFY 100k Marshfield human sex-averaged

Family	Chromosome	LOD	SNP	Position cM
99	3q13.3-13.33	2.03	rs1398748	120.04
100		2.12	rs437749	107.64
	1p31.1	2.16	rs400382	101.29
	2q31.2	2.18	rs1002207	186.26
	2q32.1	2.18	rs6754252	188.90
	2q33.3	2.12	rs7602656	201.47
	8q22.1	2.12	rs2245832	101.69
	11p15.1	2.12	rs730348	31.78

### Dominant model

No linkage peak was obtained under autosomal dominant with assumed parameters neither using Illumina 6K Linkage 24 deCODE Human Genetic Map nor AFFY 100k Marshfield Human Sex-Averaged. Family 99 was the solo family resulted in a maximum LOD score of 1.51 at rs39552 at position 114.94 cM on 16q23.3-q24.1. Linkage intervals for chromosomes 4q22.3 (HLOD = 1.84 at rs9307161, α = 0.2390, 102.56 cM) and 17q22 (HLOD = 1.5658 at rs2045418, α = 0.3396, 80.09 cM) reached HLOD ≥1 in total families.

## Discussion

### Linkage intervals

Both nonparametric and parametric analysis methods were applied to the data with an assumption of the presence of both autosomal dominant and recessive inheritance. As the sample size was not large, both types of analysis were carried out to ensure reliability and accuracy of the results. Parametric analysis with LOD score requires specified mode of transmission of all loci to avoid erroneous results. Other alternative methods of linkage analysis have been developed and are known as nonparametric. Nonparametric analysis is beneficial as it does not require specified values in the transmission model. Generally, based on linkage analysis, the most probable mode of inheritance among families is autosomal recessive. Furthermore, analysis of family pedigrees has found parent-offspring inheritance for NSCLP; 33.3 % of cases were autosomal dominant and 66.7 % were autosomal recessive. Autosomal dominant occurs when a disease allele from an affected parent is transmitted to the offspring and the offspring suffers from the disease and tends to occur in every generation of an affected family. Conversely, autosomal recessive inheritance assumes that both parents are unaffected but that both carry the disease allele. It is not likely to occur in every generation of an affected family. The overall analysis through family tree leads to a conclusion of autosomal recessive inheritance which most probably occurred since the normal phenotype could not be distinguished as a carrier or non-affected person.

The data set produced from both genetic maps was helpful in exploring larger probability of genes involved in clefting. Some produced data were found overlapped between the two genetic maps but some findings are exclusive to each genetic map. All interested findings were combined for better understanding of linkage distribution. Several linkage intervals attained the suggestive statistical threshold in NPL analysis in total families on locus 2q33.1-q35, 4q22.1-q25, 7q22.3-q33, and 14q12. However, the analysis of each family found that family 50 and family 58 attained several suggestive linkage thresholds while family 100 attained significant linkage of NPL scores on locus 1p31.1-31.3, 2q31.1-q35, 7q22.3-32.2, 8q22.1-q23.3, 11p15.3-p13, 13q22.2-q31.1, 14q12-q21.1 and 22q11.1-q11.21. Similar NPL scores was found in family 100 which means that it has higher probability of mutation harbored in larger linkage intervals since they have been intact throughout generations [23].

Parametric analysis for autosomal recessive that reached LOD score at suggestive level was found in family 99 and family100. Suggestive linkage was attained at locus 3q13.3-q13.33 in family 99 and the rest 1p31.1, 2q31.2-33.3, 8q22.1 and 11p15.1 in family 100. Nonsyndromic clefting is known to be genetically heterogenous, therefore susceptible genes may vary in each family.

### Candidate genes within suggestive and significant linkage interval

Linkage interval in the locus of 1p31.1-p31.3 has attained significant NPL and suggestive LOD score in family 100 and suggestive linkage in family 50 with HLOD = 1.73, α = 0.308 under recessive model. Positive α indicates that a subset of families might be linked to this chromosomal region. Previous study has reported that HLOD can provide robust and powerful tool for detection of linkage in the presence of heterogeneity [24]. Findings on both parameters reflect to the assumptions of higher possibility that these linkage intervals harbor a mutation. Gene of interest that found within this region was Latrophilin 2, a G-protein-coupled receptor (*LPHN2*).

Several studies have documented their findings regarding 1p chromosomal region. A novel homozygous intergenic deletion of 237-kb at 1p31 was reported in four affected siblings of consanguineous marriage and 1p31 is an autosomal recessive locus [14]. It was previously reported that locus 1p34 has a positive linkage among families with a history of Van der Woude syndrome (VWS) that brought it to the interest because it closely resembles the phenotype of NSCLP [25]. In addition, chromosomes region of 1p36 has been reported as having NPL suggestive linkage on this region but not on 1p31, which was different from our finding. None reported the role of *LPHN2* (latrophilin 2, a G-protein-coupled receptor) in 1p31 region in human craniofacial development so far. *LPHN2* is a tumor suppressor genes that was found within oral submucous fibroris [26].

The linkage interval in 2q31.1-q35 has shown one of the highest significant NPL score in total data set and in family 100 and suggestive NPL score in family 50. This chromosomal region also attained a suggestive LOD score in family 100 and HLOD score of 2.63, α = 0.124 under autosomal recessive mode of inheritance for total family. Several genes attained between 2q31.1-q35 linkage intervals may play a role in craniofacial development. However, the most common genes associated with craniofacial development were Special AT-rich sequence-binding protein 2 (*SATB2*) and Small ubiquitin-like modifier 1 (*SUMO1*). The role of *SATB2* in craniofacial development has been discussed widely. Strong expression of *SATB2* was detected during palatal shelves development with maximum expression in the mesenchyme underlying the medial edge epithelia [27]. Strong evidence emerged from multiple unrelated reports including several patients who had deletion at 2q32-33 [28–31], a patient who had a translocation with a breakpoint site in the SATB2 gene and another who had *SATB2* mutation.

Suggestive linkage of LOD score in family 99 and HLOD ≥1 in total family for 3q13.3-13.33 chromosomal region brought to the interest of this study, with the findings of Poliovirus Receptor-Related 3 (*PVRL3*) gene related to orofacial cleft. Sözen et al., [32] has screened several regions of coding exons, adjacent introns and noncoding sequences of *PVRL3* and concluded that no variant mutations found in the selected regions in Caucasian polpulations. However, there is possibility that the variants were not detected by the SSCP/heteroduplex screening method used [32]. Suggestive linkage at chromosomal locus 6p12.2 from family 58 harbor *COL21A1* (alpha chain of type XX1 collagen) that have never reported associated with craniofacial development. *COL21A1* is a member of FACIT collagen family (fibril-associated collagens with interrupted helices). Type XXI collagen is localized to tissues containing type I collagen. Therefore, like other members of this collagen family, it serves to maintain the integrity of the extracellular matrix. However, two previous studies have reported haplotype in *COL11A2* at 6p21.3 associated with nonsnyndromic cleft [33, 34] while Melkoniemi et al., [35] found it was associated with Pierre-Robin sequence, cleft palate, micrognathia and it may possibly be predisposed to nonsyndromic conditions.

The 14q12 linkage interval was significant in LOD analysis and suggestive in NPL analysis. Role of 14q in the etiology of oral clefts has been well known and this chromosome contains several oral cleft candidate genes such as *ISGF3G* at 14q11.2, *JAG2* at 14q32, *PAX9* at 14q13.3, *TGFB3* at 14q24.3 and *BMP4* at 14q22.2. A weak positive linkage autosomal recessive to the 14q12 (LOD = 1.09 at 26 cM) was found among 36 multiplex Chinese families [36]. Forkhead box G1 (*FOXG1*) and HECT domain containing E3 ubiquitin protein ligase 1 (*HECTD1*) gene found within the chromosomal region of 14q12 in family 58 and family 100, which play a role in craniofacial development. TOX High Mobility Group Box Family Member 3 (*TOX3*) found in the chromosomal region of 16q12.1. Microdeletion in this region has led to a syndromic disease [37]. It was reported that a de novo deletion on 4.7 Mb 16q12.1-q12.2 caused severe craniofacial dysmorphism and psychomotor delay [38].

## Conclusions

A high degree of heterogeneity and linkage was detected in family 50, 58, 99 and 100 from the Malay population. Significant NPL score was detected primarily on family 100 thus assuming high gene linkage in this family. The novel findings of *LPHN2* at 1p31, *PVRL3* at 3q13.3, *COL21A1* at 6p12.1 and *TOX3* at 16q12.1 and known genes *SATB2* would shed more light on the disease mechanism that has been discovered through genome-wide linkage analysis. A comprehensive investigation of the candidate genes in orofacial clefts is warranted.

### Availability of data and materials

The microarray data for the all families are available by the following doi:10.6070/H41C1TWH that was deposited in LabArchives [39].

## Abbreviations

CL: cleft lip
CLP: cleft lip and palate
COL21A1: alpha chain of type XX1 collagen
CP: cleft palate
FOXG1: forkhead box G1
FOXP2: forkhead box protein P2
HECTD1: HECT domain containing E3 ubiquitin protein ligase 1
HLOD: heterogeneity LOD
HWE: Hardy-Weinberg equilibrium
LOD: logarithm of the odds
LPHN2: latrophilin 2, a G-protein-coupled receptor
NCBI: National Center for Biotechnology Information
NPL: nonparametric linkage
NSCLP: nonsyndromic cleft lip with or without palate
PVRL3: poliovirus receptor-related 3
SATB2: special AT-rich sequence-binding protein 2
SUMO1: small ubiquitin-like modifier 1
TOX3: TOX high mobility group box family member 3
VWS: Van der Woude syndrome
